# A Deep Look Into Erionite Fibres: an Electron Microscopy Investigation of their Self-Assembly

**DOI:** 10.1038/srep16757

**Published:** 2015-11-16

**Authors:** Roberto Matassa, Giuseppe Familiari, Michela Relucenti, Ezio Battaglione, Clive Downing, Alessandro Pacella, Georgia Cametti, Paolo Ballirano

**Affiliations:** 1 Department of Anatomical, Histological, Forensic and Orthopaedic Sciences, Section of Human Anatomy, Sapienza University of Rome, Via A. Borelli 50, 00161 Rome, Italy; 2 Centre for Research on Adaptive Nanostructures and Nanodevices (CRANN), Trinity College Dublin, Dublin 2, Ireland; 3 Department of Earth Sciences, Sapienza University of Rome, Piazzale A. Moro, 5, I-00185 Rome, Italy; 4 Institut für Geologie, Universität Bern, Freiestraβe 3, CH-3012 Bern, Switzerland; 5 Rectorial Laboratory Fibres and Inorganic Particulate, Sapienza University of Rome, Piazzale A. Moro, 5, I-00185 Rome, Italy

## Abstract

The exposure of humans to erionite fibres of appropriate morphology and dimension has been unambiguously linked to the occurrence of Malignant Mesothelioma. For this reason, a detailed morpho-structural investigation through Electron Microscopy techniques has been performed on erionite samples collected at two different localities, Durkee (ED) and Rome (ER), Oregon, USA. The sample from Rome has been also investigated after a prolonged leaching with Gamble’s solution (ER4G) in order to evaluate the possible occurrence of morpho-structural modifications induced by this Simulated-Lung-Fluid (SLF). Here we report how the micrometric erionite fibres evolve in irregular ribbon- or rod-like bundles as a function of different nano-structural features. The reasons for the observed morphological variability have been explained by considering the structural defects located at ED surface fibrils (bi-dimensional ribbons) and the presence of nontronite, an iron-bearing clay mineral embedding the ER fibrils (mono-dimensional rods). ER4G shows a decrease in width of the rod-like fibres due to their partial digestion by SLF leaching, which synchronously dissolves nontronite. The reported results represent a valuable background toward the full comprehension of the morphological mechanisms responsible for potentially damage of lung tissue through the potential relocation of fibers to extrapulmonary sites, increasing the carcinogenic risk to humans.

Six minerals, five amphiboles (antophyllite, tremolite, actinolite, crocidolite, and amosite) and serpentine chrysotile, are currently regulated as “asbestos”^1^,^2^. Nevertheless, erionite, a zeolite characterised by fibrous morphology, is currently categorized, like “asbestos”, as a Group I carcinogen by the International Agency for Research on Cancer (IARC^3^,). The toxicity and carcinogenic potential of erionite, which largely exceed those of asbestos^4^, is associated with its *in vivo* durability, respirable size, and very high surface area. Epidemiologic studies on the toxicity of erionite were carried on in three villages located in the Cappadocian region of Central Anatolia in Turkey, where erionite was used in construction material^5^. People in these villages showed significantly increased percentages of MM and other outcomes associated with asbestos exposure, including localized and diffuse pleural thickening and interstitial fibrosis^5^,^6^,^7^,^8^. Moreover, a high incidence of Lung Cancer and Malignant Mesothelioma (MM) linked to erionite fibre exposure was demonstrated in a rural community in Central Mexico^9^, where environmental exposure to erionite is the main cause of the high rates of MM mortality. Recently was discovered that erionite induces production of autoantibodies and IL-17 in C57BL/6 mice^10^, in fact both erionite and amphibole asbestos induce autoimmune responses in mice, suggesting a potential for adverse effects in exposed communities. Although fibrous zeolite deposits are common in the Intermountain West region of the United States (U.S.) including areas of Nevada, California, Arizona, Colorado, Idaho, New Mexico, North Dakota, South Dakota, Utah, and Wyoming^11^,^12^, only a few studies of erionite exposure have been performed so far. Following the discovery of erionite in samples of road dust near Battle Mountain, Nevada a review of chest radiographs (n = 275) from a local community hospital was conducted. In this study the prevalence of pleural plaques was found to be 1.8% with no pleural calcifications identified^13^. Over the past few decades, gravel pits have been excavated in areas where naturally occurring deposits of erionite are found and the gravel used to surface local roads, parking lots, and other areas^14^. Ambient and activity-based air sampling and indoor dust sampling conducted by the U.S. Geological Survey (USGS) and the U.S. Environmental Protection Agency (USEPA) has confirmed the presence of erionite in gravel in some of these areas^15^. Exposure to commercial asbestos occurs primarily in occupational settings. In contrast, exposure to erionite is worldwide reported mainly through environmental pathways.

Erionite pertains to the so-called ABC-6 family^16^, whose members may be described as built-up from the stacking along the **c**-axis, following an ABC scheme, of layers of six-membered rings of (Si,Al)O_4_ tetrahedra. It is characterized by a six-layer repetition. Of the 10 different stacking sequences possible for a period of six-layers, allowing for the occurrence of both single- and double-rings, only four correspond to known minerals: AABAAC, erionite^17^, AABBCC, chabazite^18^, ABBACC, bellbergite^19^, and ABABAC, liottite^20^. In the recent years, a few papers have been published aimed at the crystal chemical and structural characterisation of erionite samples^21^,^22^,^23^,^24^,^25^ in order to provide a sound background for chemical reactivity investigations. In fact, a part of morphological reasons, toxicity of erionite has been partly ascribed to the effect of ion-exchanged iron, imbibed after inhalation, arising from protein injury^26^. Therefore, iron may induce DNA damage participating to Fenton chemistry^8^,^27^,^28^,^29^. Although chemical analyses of several erionite samples revealed the presence of iron^30^,^31^, it has been demonstrated that it was located at the zeolite surface as iron-oxide nanoparticles^21^ or as thin coating of iron-bearing phyllosilicates^22^,^24^,^25^.

Several morphological studies of erionite fibres have been carried out since the 50’s, using different electron microscopy techniques^17^,^30^,^32^,^33^,^34^. However, they lack the required level of detail necessary for supporting the attempts to evaluate the dependence of the biological activity of erionite from its microstructural features, as for example nanostructure of self-assembled aggregates. In order to fill this gap, present work aims at characterising, at the single particle level, two samples of erionite, identifying differences in morphology and self-assembly of the fibres that was found to consist of flaky, flat fibrous, or rod-like bundles. Such variability has been related to the nanostructural features of the fibrils aggregating to form different morphologies of bundles. The different dimensions of the fibrils, coupled with the observed extended defectivity, are responsible for the different mechanical behaviour of the two analysed samples (flexibility instead of brittleness). Present results, indicate that particular attention should be paid for properly evaluating the toxicological effects related to the dimension of the erionite fibres, as well as to their malignant tendency to split, as the latter feature may be potentially crucial to significantly modify their surface area and, accordingly, their reactivity. Moreover, the presence of extended surface defects is especially suited for providing sites that are able to fix cations as, for example, Fe^3+^, that has been identified as DNA-damaging agent^28^. Finally, a further sample, leached in a Simulated Lung Fluid (SLF), was analysed for investigating nanoscopic changes possibly induced by the prolonged contact with the SLF.

## Results and Discussion

### Morphological analysis

**Figure 1:**
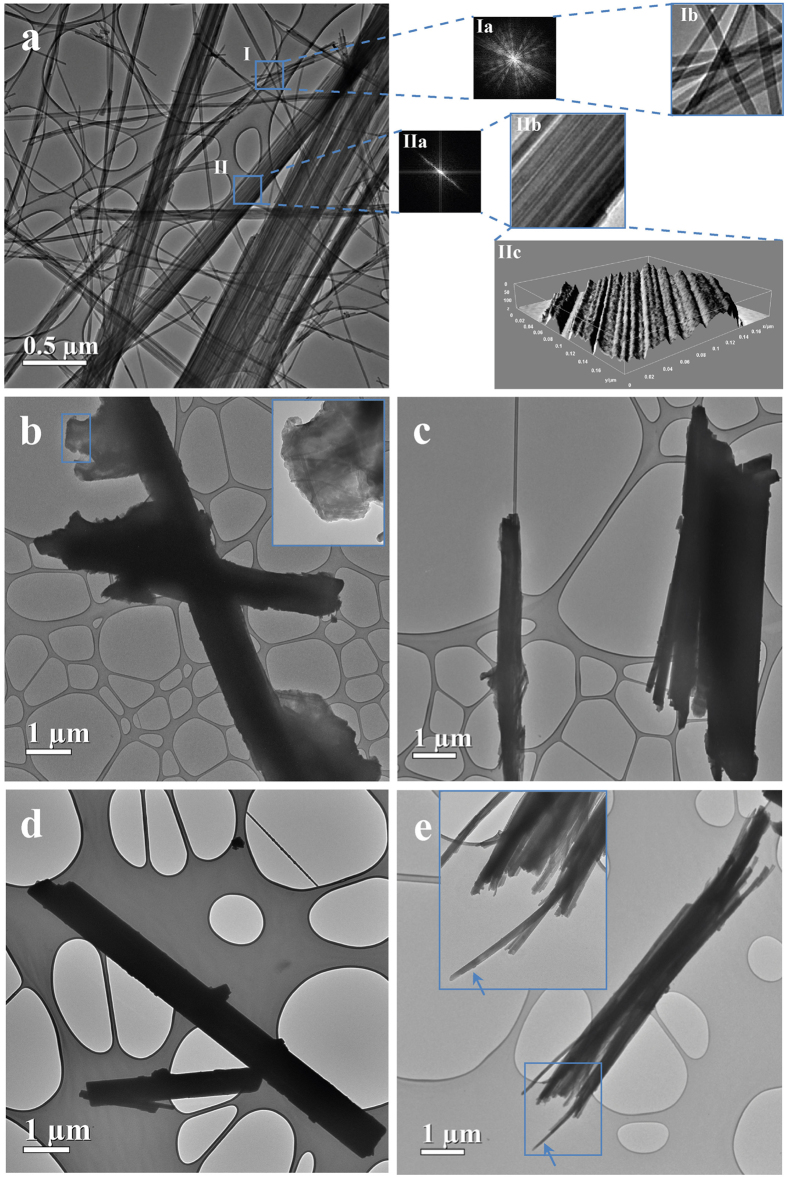
Morphometric study of ED, ER, and ER4G fibres. (**a**) BF TEM image of ED fibres aggregates. Blue squares identify the investigated region I and II. Panel Ia: FFT pattern of region I showing several radial sharp lines with different orientations. Panel Ib: IFFT image of Fig. Ia showing the disordered organization of fibres. Panel IIa: FFT pattern of region II showing a single radial broad line. Panel IIb: IFFT image of Fig. IIa showing aligned fibres in ribbon-like aggregation. Panel IIc: 3D interaction surface plot obtained from Fig. IIb. (**b**) BF TEM image of ER fibres. Inset: flaky material embedding isolated fibrils. (**c**) BF TEM image of ER fibres showing exfoliated fibrils. (**d**) BF TEM image of ER4G fibres. (**e**) BF TEM image of ER4G fibres. Inset: fibres splitting.

Despite significant research effort on the morphological investigation of asbestos fibres over the last century, studies related to erionite are, by far, less detailed. Therefore, a thorough and accurate characterisation of the three erionite samples has been undertaken for establishing the self-assembly behaviour of the fibrils as a function of their dimensional variability. Figure 1 reports representative TEM micrographs of three erionite samples, depicting the morphological heterogeneity of the fibres, drastically changing in packing density and dimension, and passing from apparently sharp, smooth to large, rough surfaces.

*Erionite from Durkee.*  Figure 1a shows typical aggregates of the woolly erionite sample ED, which are characterised by different widths, varying from ca. 25 nm to ca. 680 nm. They consist of fibrils with an average diameter of ca. 16 nm, which aggregate parallelly. Such morphological features agree with those previously reported, from FE-SEM surface analysis, by Cametti *et al.*^25^. Fast Fourier Transform (FFT) live scanning has been applied to evaluate the self-assembling ability of the fibres of aggregating in a regularly aligned bundle at micrometric scale. A selected FFT of **region I** (blue square), highlighted in Fig. 1a and covering an area of approximately 0.25 × 0.25 µm^2^, shows several radial sharp lines with different orientations testifying a three-dimensional stacking of the fibrils (**Panel Ia** of Fig. 1a). This area discloses, at a nanometric scale, the entangled arrangement of fibrils responsible for the macroscopic woolly aspect of the sample. The disordered grouping of the fibrils is shown in **Panel Ib,** obtained by Inverse FFT (IFFT) of **Panel Ia**. A very interesting self-assembly behaviour is highlighted by the FFT image of **region II**, in which only a fairly broad line occurs, indicating that all fibrils are perfectly aligned (**Panel IIa**). The corresponding IFFT displays one relatively large aggregate characterised by a width of 200 nm in which parallel well-aligned fringes are observed (**Panel IIb**). Moreover, a 3D interaction surface plot of **Panel IIb** has been reported for visualizing the surface features of the bundle (**Panel IIc**). By monitoring the contrast intensity, it is possible to deduce that the thickness along the direction perpendicular to the surface plot (**z**-axis) slightly increases from left to right. This morphological feature indicates that fibrils form micrometric aggregates with irregular surface and thickness leading to ribbon-like bundles, instead of rod-like ones, in which the contrast intensity is higher at the centre than at the edge (See also in Supplementary Informations Fig. S1).

The Energy Dispersive X-ray (EDX) spectrum acquired on the ribbon-like bundle of **region II** revealed, in agreement with Ballirano *et al.*^22^, the occurrence of Na, Mg, Ca, O, K, Al, and Si chemical species, as reported in Supplementary Information (Fig. S2a).

*Erionite from Rome.* Unfortunately, it was impossible to use the accelerating voltage of 80 kV for investigating the structural features of the ER sample because of the occurrence of flaky material adhering to the surface of the fibres, limiting the bulk observations. In fact, imaging required a longer exposure time, eventually favouring the production of artefacts originating from beam damage that affected the quality of the morphological information^33^. To overcome this experimental problem, a lower accelerating voltage @ 60 kV has been used to probe both ER and ER4G samples.

The ER sample consists of thick and rough fibres of ca. 1.2 μm in diameter, as well as flaky aggregates, attached to the edge of the fibres, are clearly visible, as shown in Fig. 1b. A magnified image (**Inset**) shows isolated fibrils of about 20 nm in diameter embedded in the flaky aggregate. This peculiar kind of embedding of erionite fibrils has been here observed, to the best of our knowledge, for the first time. EDX spectra of the ER sample, used to probe the area shown in the **Inset**, revealed approximately the same chemical composition of ED, but in addition small amounts of Fe were detected (Fig. S2b), consistently with reference data^21^,^30^,^31^.

Other microscopic areas of the same sample were investigated to improve the micrometric observations of the sample gathered from local TEM technique, as shown in Fig. 1c (See also in Supplementary Informations Fig. S1). The diameter of the large fibres, with rod-like shape, spans from about 0.50 to 1.15 μm in diameter being much thicker at the centre than at the edge. Differently from the micrometric area of Fig. 1b, splitting (cleavage) and fraying occurs in fibres with diameters smaller than 300 nm, aggregating into large ones. Contrarily from the ED sample, well-separated or isolated fibrils were not found, probably due to the amount of flaky material surrounding the single fibril, which may additionally act as a cementing medium preventing splitting.

*SLF leached erionite from Rome.* The ER4G sample (Fig. 1d) shows, similarly to ER, isolated thick fibres of about 0.5 and 1.0 μm in diameter with adhering short fibres of ca. 100 nm in width. The absence of the flaky aggregates sticking to the edge of the fibres, originally occurring in untreated ER, is reasonably related to the leaching process. EDX analysis of fibres indicated the absence of iron confirming the findings of Ballirano and Cametti^24^ (Fig. S2c). Following the same approach used for ER sample, after accelerating voltage reduction, other areas of the sample were investigated disclosing the occurrence of thick single fibres of ca. 1.0 μm with split ends, as displayed in Fig. 1e (See also in Supplementary Informations Fig. S1). Besides, a partially exfoliated bundle, showing single fibrils of ca. 50 nm in diameter and some fractured fibres, can be also noticed in the **Inset**. Furthermore, it should be noted that a progressive increase of the bending of the curled fibrils, occurring in the fibre bundles with split ends (Fig. 1e, blue arrows), has been observed during the measurement, testifying the high sensitivity of erionite under the electron beam.

The morphometric investigation, carried out on the various erionite samples, provides different relevant information. The ED sample consists of fibrils aggregating in ribbon-like bundles that show high variability of width, which spans from 25 to 680 nm (see Table S1 in Supplementary Information). Fibrils of 16 nm in width, clearly visible in the TEM images, tend to coalesce forming parallel arrangements leading to irregular flat ribbons. Conversely, the ER sample shows completely different fibres morphology, consisting of rod-like bundles of fibrils, irregularly aligned, and surrounded by flaky aggregates that partially prevented the visualization of the single fibril. The diameter of the thickest rods ranges from approximately 0.5 to 1.2 μm, while the smallest ones span from 150 to 300 nm. Small and isolated fibrils showing a diameter of about 20 nm, similarly to ED, have been only detected within the flaky material. Therefore, the dimensional features of ED have an opposite behaviour with respect to ER. In fact, the width of the ED ribbon-like bundles shows a variability of one order of magnitude greater than that of the ER rod-like fibres. On the contrary, the diameter of the ED small fibrils is very constant, differently from the high variability of the ER fibrils, as reported in Table S1. The ER4G sample is characterised by a slight reduction of the fibres diameter as compared to ER, probably because of partial dissolution occurring during the leaching processes. Those findings agree with the crystallite size^35^ reduction after leaching determined by XRPD^24^. As a further result of the leaching effect, a complete removal of the flaky material surrounding the fibres has been observed.

### Structural analysis

To gain a deeper insight into the self-assembly behaviours, which drive the morphological differences shown among the samples, a detailed structural investigation at nanometric scale has been performed.

**Figure 2:**
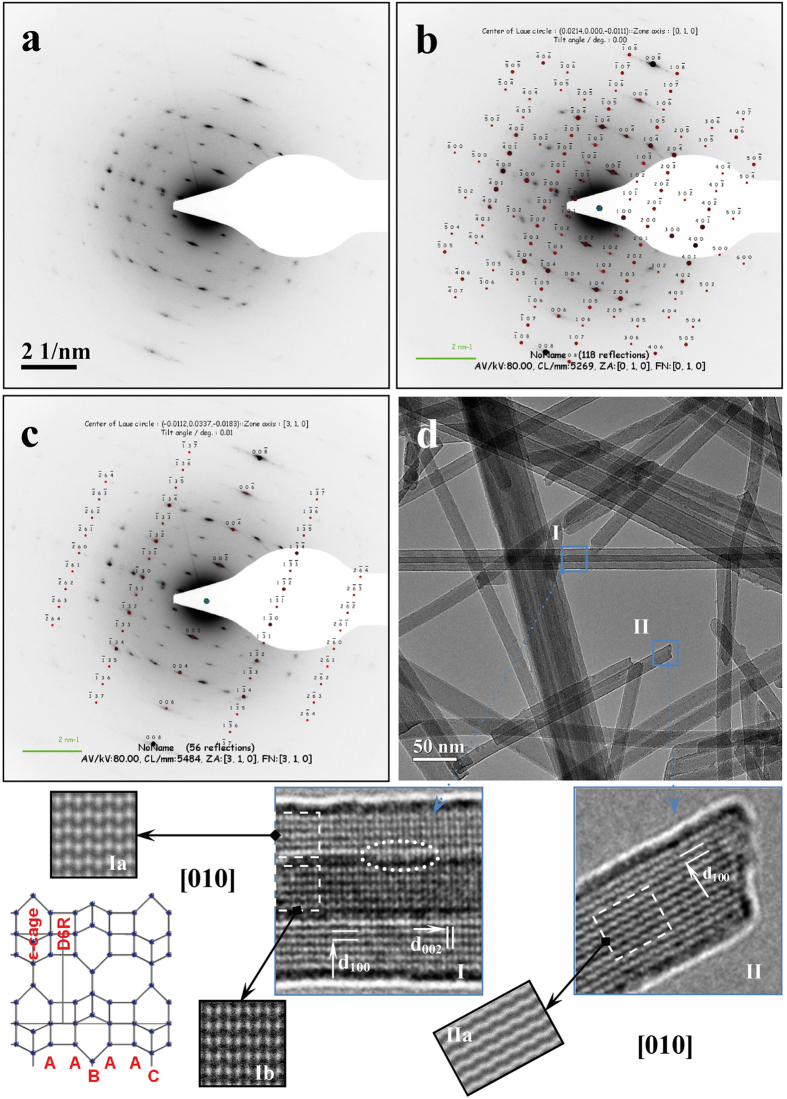
Structural study of ED fibres. (**a**) EDP taken from region II of Fig. 1a, showing a complex regular rectangular array of diffraction spots. (**b**) Simulated EDP of Fig. 2a produced by erionite fibres oriented along the [010] axis. (**c**) Simulated EDP of Fig. 2a produced by erionite fibres oriented along the [310] axis. (**d**) BF TEM image of fibres aggregates of the ED sample. Blue squares identify the investigated region I and II. Panel I: Magnified area of region I showing two overlapped fibrils of different width. The corresponding *d*-spacings of the visible lattice fringes are marked by white arrows. Panel Ia: Simulated image of the bright lattice fringes zone, marked by a white-dotted square in Panel I, of erionite as seen along the [010] axis. Sketch: Atomic representation of the erionite structure along the [010] axis. The AABAAC stacking sequence of planes of six-membered rings of (Si,Al)O_4_ tetrahedra is also indicated. Panel Ib: Simulated image of the dark lattice fringes zone, marked with a white-dotted square in Panel I, of erionite as seen along the [010] axis. Panel II: Magnified area of region II showing a single fibril. The corresponding *d*-spacing of the visible lattice fringes is marked with a white arrow. Panel IIa: Simulated image of the bright lattice fringes zone of the nanometric area marked with a white-dotted square in region II as seen along the [010] axis.

*Erionite from Durkee.* The area of sample ED **(region II** of Fig. 1a) has also been characterised by electron diffraction technique in order to establish its crystallographic features at nanometric scale^36^. The corresponding Selected Area Electron Diffraction (SAED) pattern shows a complex, albeit regular rectangular array of diffraction spots and, along one radial direction, high order intense reflections with diffraction diffuse streaks can be noticed (Fig. 2a). A simulation of the electron diffraction pattern (EDP) has been performed for investigating the relative orientation of the fibrils using the structural data of erionite retrieved from Ballirano and Cametti^20^. The refined hexagonal cell parameters (space group *P*6_3_/*mmc*) are consistent with those of reference data. The best theoretical analysis and indexing has been obtained by considering two overlapped EDPs, corresponding to [010] and [310] zone axes parallel to the incident electron beam (Fig. 2b,c, respectively), produced by two differently oriented fibrils occurring within the ribbon. Accordingly, one fibril is rotated with respect to the other of ca. 79° around the [001] direction, which is parallel to the ribbon elongation. The appearance of some slightly curved streaks indicates that fibres are imperfectly aligned at nanometric scale.

Unfortunately, High-Resolution (HR) TEM investigation was unsuccessful at providing direct structural information because of the above-mentioned instability of erionite under the electron beam. Indeed, fibre damage occurred as the magnification was increased for reaching HR conditions. Fig. S3 reports an example of such severe damage. The magnified **region I** of Fig. S3a clearly shows lattice fringes within the fibrils that disappear as magnification increases (Fig. S3b). Such process is coupled with fibril bending. To avoid the occurrence of any fake structural information, we were forced to perform fast data acquisition at medium magnification using the minimum dose method. Nevertheless, this experimental set up was perfectly suited for visualising lattice fringes as a result of the relatively large value of the relevant *d*-spacings of erionite (>0.7 nm) (Fig. 2d, See also in Supplementary Informations Fig. S1). The investigated area of the ED sample shows self-aggregating fibrils forming small ribbons that span in width from ca. 26 nm of two partially overlapped fibrils, up to ca. 69.6 nm of larger aggregates. Accurate measurement of the single fibril diameter, using the *d*_100_ lattice fringes of erionite for image calibration, provided values comprised between 15.5 and 16.5 nm with a mean value of 16.0 nm. Hence, we were able to establish that the common overlapped width between two aligned fibrils has a value of ca. 6 nm. Moreover, the calculated dispersion of ± 0.5 nm around the width mean value of 16 nm, corresponding approximately to 14 unit cells, can be possibly related to different amounts of surface defects.

The Bright Field (BF) TEM image shows elongated fibrils stacked in a disorderly way, coupled to a decrease of the mean ribbon width. The selected magnified nanometric area referred to as **region I**, contains two fibrils unusually aligned, one characterised by a width of 20 nm clearly showing lattice fringes, and a second one, having a diameter of ca. 10 nm, characterised by darker lattice fringes, apparently superimposed on it (**Panel I** of Fig. 2d). A qualitative evaluation of the image suggests that fibrils could be considered adjacent and not pertaining to a concentric arrangement (e.g. multiwall nanotube), because Fresnel fringes are visible around the edge of the inner fibril (indicated by the white-dotted ellipse). This diffraction phenomenon indicates that each fibril has a different height parallelly to the electron beam. A detailed analysis of the image clearly shows rough surfaces at the edge of the fibre indicating a fairly abundant occurrence of structural defects. Two different *d*-spacings have been identified and measured. The *d*-spacing perpendicular to the long axis of the fibre corresponds to *d*_100_ = 1.148 nm, while the *d*-spacing parallel to the fibre elongation corresponds to *d*_002_ = 0.747 nm (both indicated with white arrows). Such values agree with those of erionite-Na listed in PDF 70-0540. Due to the structural complexity of erionite, the image has been processed by simulation. The simulated image of the corresponding nano-area (white square) has confirmed the orientation of the fibril along the [010] zone axis with thickness of about 18.2 nm, as shown in **Panel Ia**. The experimental image consists of broad and noisy bright and dark patches, which was reproduced by considering a defocus values of -95.5 nm away from Scherzer defocus (i.e. underfocus image). Comparison between the skeletal representation of the erionite framework along the [010] zone axis (only Si,Al atoms shown), displayed below **Panel Ia**, and the simulated image permits to attribute the intense bright patches to cancrinite ε-cages and the bright lines to D6R rings. Image simulations indicate that the framework is terminated with the cancrinite ε-cage on the side-wall of the fibre (**Panel I** of Fig. 2d), consistently with the findings of Ohsuna *et al.* for zeolite-L^37^. It is worth noting that the dark patches correspond to both the opening of the large erionite cages (boat-shaped 8 membered-ring 8MR) or, possibly, to the cross-section of the cages, which delimitates mesopores. The exposition to the surface of such terminations maximizes both ion-exchange properties and surface area justifying the extreme reactivity of erionite fibres.

By decreasing the defocus and increasing the thickness parameters of the simulated image at values of –136.5 nm and 27.5 nm, respectively, the contrast intensity increased without significantly altering the motif of the bright and dark patches, thus obtaining a remarkably good simulated image of **Panel Ib**. The differences in both defocus and thickness quantitatively indicate that fibrils are stacked parallelly to the [001] direction in agreement with the observed Fresnel fringes at the edge.

Besides, the top-termination of a single fibril, referred to as **region II** (**Panel II**), characterized by a width of ca. 16 nm, has been investigated. Within this region, lattice fringes corresponding to *d*_100_ were identified. The image has uniform contrast intensity, showing a zig-zag pattern, which is different with respect to the alternating up and down distribution of the bright and dark patches pattern observed in **Panel I**. The image was simulated using as starting parameters those obtained from **Panel Ia**. The calculated image displayed in **Panel IIa**, obtained for a specimen thickness of ca. 14.3 nm and an orientation of the atomic packing along the [010] zone axis, well matches the experimental one. By a further decrease of the defocus value, a zig-zag pattern appeared at a defocus value of −175 nm. Both simulations performed on two different fibrils images provided a thickness slightly smaller than the measured width. This observation indicates that the cross section of the fibre has quasi-ellipsoidal instead of cylindrical-like shape possibly due to the combined occurrence of both {hkĪ0} di-hexagonal and {10ĺ0} hexagonal prisms as delimiting crystal forms of the fibril.

A hexagonal cross-section should favour honeycomb-like packing, which could evolve also in a rod-like shape at micrometric scale (e.g. bundle of carbon nanotube). Instead, our morphometric investigation clearly indicates a prevailing ribbon-like shape of irregular thickness.

Several morpho-structural features are responsible for the loss of regularity of the shape of the fibril leading to a ribbon-like bundle. Accurate evaluation of TEM images highlights that neighbouring stacked fibrils exhibit a tendency to laterally superpose instead of being attached by edge as in the case of fibrils with circular cross-section. This morphological behaviour can be related to their pseudo-hexagonal cross-section, which favours an interfacial contact acting via the faces of hexagonal (laterally more extended) and di-hexagonal (laterally less extended) prisms of two adjacent fibrils.

This hypothesis is experimentally confirmed by observing that the prevailing orientation along the [010] direction, disclosed by images, should result in regular honeycomb-like packing. Instead, EDP analysis revealed the occurrence of a few fibrils oriented along [*hk*0] directions within the same ribbon. Therefore, the stacking of differently oriented fibrils favours the formation of a ribbon-like aggregate with irregular thickness following the mechanism above described. Moreover, the occurrence of structural defects at the edge of the fibril, aligned along the (100) surface, are a further feature acting to favour the evolution of the aggregation toward a bi-dimensional growth of a micro-ribbon shape. No stacking faults of offretite type AAB^34^,^38^, perturbing the regular AABAAC sequence of erionite, were observed in the present sample.

**Figure 3:**
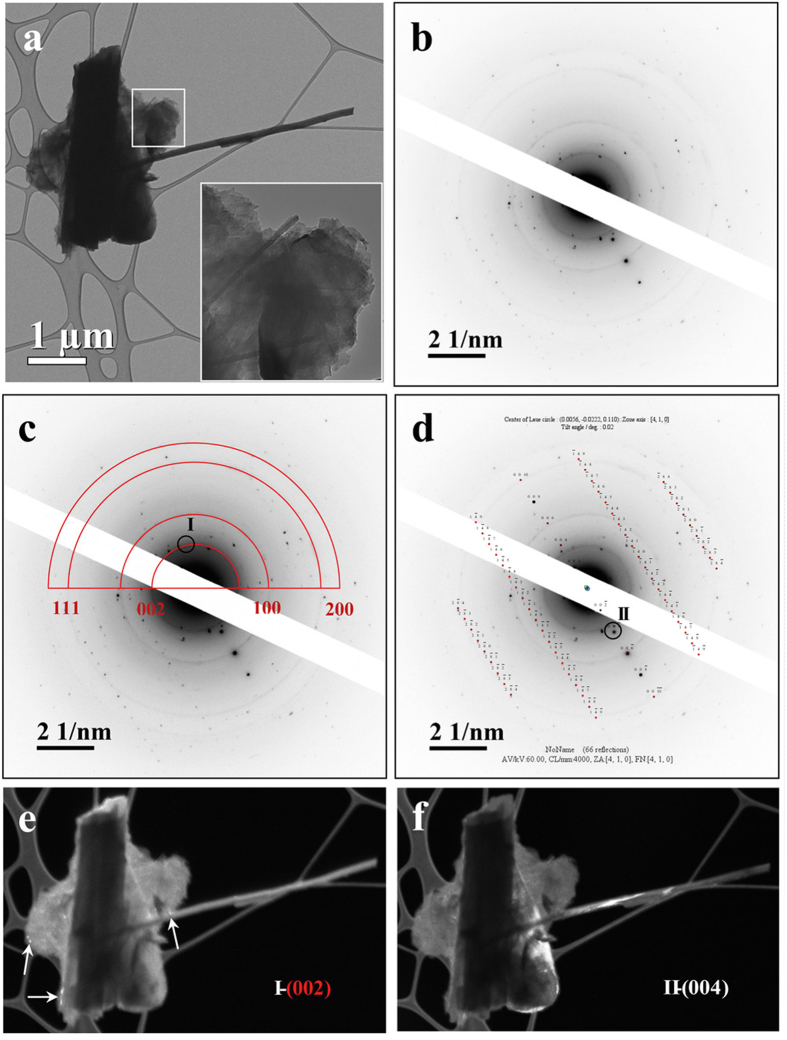
Structural study of ER fibres. (**a**) BF TEM image of ER micro-fibre. Inset: flaky material embedding isolated fibrils. (**b**) EDP taken from Fig. 3a shows an overlapped contribution of a complex array of diffraction spots and diffraction rings. (**c**) EDP of Fig. 3b showing distinct diffraction rings corresponding to polycrystalline nontronite (red arcs). (**d**) Simulated EDP of Fig. 2a showing fibres of erionite oriented along the [410] axis. (**e**) DF TEM image of Fig. 3a obtained by selecting the micrometric area of the nontronite (002) reflection, identified by a black circle in Fig. 3c. White arrows point to small nontronite particles. (**f**) DF TEM image of Fig. 3a obtained by selecting the micrometric area of the erionite (004) reflection, labelled by a black circle in Fig. 3d.

*Erionite from Rome.* Figure 3a shows two thick and short fibres of about 1.0 μm of diameter crossing each other, with two parallel fibrils of 100 nm of diameter completely engulfed by a flaky particle (See also in Supplementary Informations Fig. S1). At relatively high magnification (Inset of Fig. 3a), it is still possible to observe the occurrence of fibrils with a diameter of 20 nm embedded in the same flaky material. Besides, SAED patterns of the complex aggregate of Fig. 3a were collected. The EDP reported in Fig. 3b shows an overlapped contribution arising from various particles. The diffraction rings, produced by a randomly oriented polycrystalline material, have been attributed to nontronite, an iron-bearing smectite of general formula (Ca_0.5_,Na)_0.33_Fe^3+^_2_(Si_3.67_Al_0.33_)O_10_(OH)_2_·nH_2_O^39^, by superposition of the corresponding *d*-spacings (red arcs of Fig. 3c) retrieved from PDF 34–0842. This clay mineral has been previously identified by X-ray powder diffraction technique on the same sample^22^. The occurrence of nontronite has also been confirmed by a qualitative EDX spectrum in which the fluorescence peak of Fe, and to a lesser extent of Ca, is clearly evident (Fig. S2b). The second series of diffraction effects, produced by erionite phase, has been indexed by EDP simulation using the same crystallographic parameters employed for the ED sample. Fig. 3d shows that several fibres are oriented with the [410] axis parallel to the electron beam. Therefore, the stacking of the ER single fibres has morphological features similar to those of the ED sample. Unfortunately, it was impossible to identify the remaining few extra-spots by simulation because of the poor organization of the diffraction effects arising from the flaky particle embedding the fibre. To visualize the spatial distribution of the different crystalline phases detected by EDP, Dark Field (DF) imaging was used. DF images have been obtained by placing the objective aperture around a diffracted beam of the EDP of region I (black circle of Fig. 3c), corresponding to the arc of the (002) Debye ring of nontronite. DF image proves that the flaky aggregate consists of polycrystalline nontronite, completely embedding the fibre. The dimensions of the small, intensely bright areas occurring within the nontronitic material are consistent with crystallite aggregates smaller than 50 nm (white arrows), as shown in Fig. 3e. Similar DF imaging process was applied for locating erionite within the aggregate by placing the objective aperture around its (004) reflection of the EDP image of Fig. 3d. DF image of Fig. 3f clearly shows bright lines along the fibre axis of both the large and small fibres. ER fibrils are close-packed in rod-like bundles of micrometric dimension, but the structural features are consistent with those of the honeycomb packing established for ED sample. Hence, we should expect the occurrence of ER under the form of honeycomb packing or a micro-ribbon arrays of fibrils. Differently, the presence of the flaky materials adhering to/embedding ER influences the evolution of the fibrils in their parallel self-assembling at micrometric scale. Furthermore, ED fibrils show evident structural defects localized on the (100) plane, while flaky nontronite surrounding ER fibres provide further defectivity around the fibre surface. Therefore, the lateral growth of the ER fibres evolves around surface of the single fibril to form a mono-dimensional rod, preventing a two-dimensional growth eventually leading to a ribbon-like shape.

**Figure 4:**
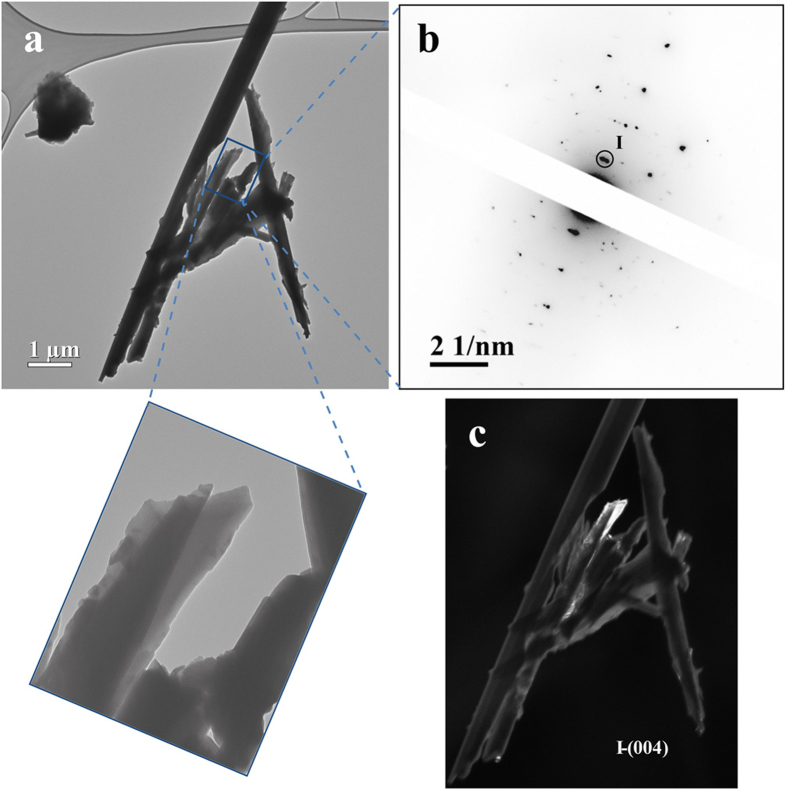
Structural study of ER4G fibres. (**a**) BF TEM image of ER4G fibres. Bottom Inset shows a magnified area. (**b**) EDP taken from Fig. 4a showing a complex array of diffraction spots and Debye rings. (**c**) DF TEM image of Fig. 4a obtained by selecting the micrometric area of erionite (004) reflection, identified by the black circle in Fig. 4b. White arrows point to small nontronite particles.

*SLF leached erionite from Rome.* The BF image of the ER4G sample (Fig. 4a) shows aggregates of smooth fibres, with diameters varying from 300 to 650 nm (See also in Supplementary Informations Fig. S1). Compared to the untreated ER sample, the diameter of the fibres is slightly smaller and the amount of nontronite flakes seems to be strongly reduced. Therefore, small fibres suffer from cleavage effects, testified by fractures and fraying of the brittle fibres observed in the magnified image (**Inset**). As previously indicated, EDX analysis of fibres indicated the absence of iron due to the removal of nontronite from their surface produced by the SLF leaching process. To identify the various mineral phases, EDP has been acquired, showing the rectangular array of diffraction spots, attributed to erionite, previously observed for the other samples (Fig. 4b). No diffraction contribution of nontronite was detected. To visualize the relative crystallographic orientation of fibres, a DF image has been acquired by placing the objective aperture around the (004) reflection of erionite. The image shows a few bright areas, indicating fully crystalline regions, of the fibres displayed in Fig. 4c. In particular, it is noteworthy that the termination of the single fibre, which has a width of ca. 300 nm, is crystalline, differently from the remaining area of the field of view, which is not bright. This result agrees with the partial amorphisation of the leached fibres reported by Ballirano and Cametti^24^ from XRPD data. Isolated nontronite flakes, reported in Supplementary Information (Fig. S4a), have been detected in other areas. Nontronite still evidences its polycrystalline behaviour as testified by the Debye rings, marked by red arcs, observed in SAED patterns (Fig. S4b). The corresponding DF image still shows a random distribution of small nontronite nanoparticle within the flake (Fig. S4c).

## Conclusions

A detailed morpho-structural investigation, both at the micron- and at the nano-scale, has been performed on erionite samples collected at two different localities, Durkee and Rome, Oregon, USA. The sample from Rome has been also investigated after a prolonged leaching with Gamble’s solution, a SLF, in order to evaluate the occurrence of morpho-structural modifications possibly induced by the treatment.

To get an insight into the self-assembly of erionite fibres at the micro- and atomic-scale, TEM imaging coupled with EDX, SAED and DF techniques also supported by appropriate imaging analyses have been exploited, paying special attention to the high-sensitivity of the fibres to the electron beam. The approach here used for investigating the self-organization of the fibrils, evolving in ribbon- or rod-like bundles, provides a direct experimental evidence of the self-assembling mechanisms.

TEM analyses, scanned on different micro-areas of the erionite samples, have evidenced the occurrence of fully crystalline fibrils mainly oriented along the [010] zone axis, less commonly along [*hk*0] directions. This morphological behaviour has been related to the occurrence of structural defects located at the surface of the fibrils, which favour the interfacial interactions and their mutual orientation in a parallel way. ED sample clearly evidences the presence of defects, mainly located on the (100) plane of the hexagonal fibrils, favouring a ribbon-like aggregation. Conversely, ER fibrils aggregate to form rod-like bundles, although their structural features are similar to those of ED. The significant difference between ED and ER is represented by the presence of nontronite flakes surrounding the surface of the ER fibrils. This clay mineral may be considered as the precursor of erionite^40^ with which shares the planes of six-membered rings of (Si,Al)O_4_ tetrahedra as building unit. Nontronite gives rise to domain defects around the surface of the single fibril, favouring the increase of the diameter of the fibre leading to rod-like aggregates. Actually, it has been highlighted that ED sample shows the smallest fibrils with a remarkably constant width of ca. 16 nm, while the ER crystalline fibrils are of ca. 50 nm or more in diameter, which can extend for hundreds of nanometer, as shown by DF image technique. It is worth noting that the different dimensions of the fibrils, coupled with the extended defectivity, are responsible for the different mechanical behaviour of the ED and ER samples (flexibility vs. brittleness). Therefore, particular attention should be paid to properly evaluate the toxicological effects related to the dimension of the erionite fibres, as well as to their malignant tendency to split, as the latter feature may be potentially crucial to significantly modify their surface area and, accordingly, their reactivity.

ER4G shows similar morphology compared to ER, but the diameter of the fibres is slightly smaller due to their partial digestion and to the complete removal of the embedding nontronite. DF images confirm the partial amorphisation of the fibres supporting the results of Ballirano and Cametti^22^. This feature could possibly reflect some role for understanding in detail the mechanisms responsible for carcinogenicity of erionite, which has been ascribed to the ability of erionite to fix iron imbibed after inhalation^26^. In fact, the presence of extended surface defects is especially suited for providing sites that are able to fix cations as, for example, Fe^3+^, that has been identified as DNA-damaging agent^28^. Differently, a very recent investigation has unambiguously proved that Fe^2+^ is ion-exchanged and hosted by the erionite cage^22^. As a final remark, present results suggest that the role of nontronite has to be investigated in detail. In fact, erionite may act as a mechanical carrier of this iron-bearing clay mineral within the human body. It is worth noting that nontronite is a common component of the Cappadocian rocks where erionite fibres are found and are unambiguously linked to the occurrence of MM. Nontronite seems to act as a cementing medium within the aggregates of erionite fibres, whenever occurring. However, leaching of fibres with Gamble’s solution, which simulates the interstitial fluid deep within the lung, detaches nontronite from the surface of the fibrils easing their splitting. It is unclear whether dissolution of nontronite occurs, as isolated flakes of the phyllosilicate were still found in ER4G. Under such respect, Ballirano *et al.*^22^ reported no Fe release from ER after 1 h in water at pH 5, but this is a very short time as compared to the typical permanence period of fibres within the lung. Reference data seem to point out that zeolites should dissolve more rapidly than clay minerals under most pH conditions^41^. However, nontronite represents one of the most stable clay mineral^42^, whereas erionite is reported as a relatively unstable zeolite^43^. Therefore, a detailed analysis of the dissolution rate of both mineral species under an extended pH range is required. Finally, it should be reminded that iron-bearing smectite shows a very relevant surface chemical activity, which depends on the oxidation state of structural iron, as indicated by the several investigations aimed at studying the smectite-mediated degradation of agricultural chemicals^44^,^45^.

## Materials and Methods

Three samples of erionite were analysed. The first one (ED) is the so-called woolly erionite-Na from Durkee, Oregon, USA; which has been recently characterised by Field Emission-SEM (FE-SEM) and X-ray powder diffraction (XRPD)^22^,^25^. The second one (ER) is erionite-Na from Rome, Oregon, USA. This fibrous sample, of sedimentary origin, has been found to show significant chemical variability, as its composition ranges from erionite-Na to erionite-K^21^,^24^. Finally, a third sample (ER4G), consisting of fibres of ER leached for 4 months in Gamble’s solution, a SLF, has been investigated. This sample has been previously characterised using SEM and XRPD techniques by Ballirano and Cametti^24^. The three samples have limited chemical differences mainly related to the presence of Ca in ED, which is absent in ER and ER4G. Moreover, ED has been shown to consists of nearly pure erionite fibres, whereas EG contains minor impurities of iron-bearing clay minerals^24^. However, the latter is available in fairly large amounts and for this reason has been used in the past, without an adequate characterisation, for chemical reactivity and cellular testing investigations. The relevant morphological differences between ED and ER are related to their different crystallisation processes. In fact, woolly erionite has been reported to be the result of low-temperature hydrothermal solutions dissolving locally abundant sedimentary erionite and redepositing it in cavities and seams in adjacent rhyolitic rocks^16^.

TEM images and Electron Diffraction Patterns (EDP) were captured using a FEI-Titan equipped with an EDAX Energy Dispersive X-ray (EDX) spectrometer, operating @ 80 keV; whereas low voltage experiments were performed using a ZEISS EM10 TEM operating @ 60 keV.

## Additional Information

**How to cite this article**: Matassa, R. *et al.* A Deep Look Into Erionite Fibres: an Electron Microscopy Investigation of their Self-Assembly. *Sci. Rep.* **5**, 16757; doi: 10.1038/srep16757 (2015).

## Supplementary Material

Supplementary Information
