# Supplementary material for: A Deep Look Into Erionite Fibres: an Electron Microscopy Investigation of their Self-Assembly
Source: Sci Rep. 2015 Nov 16;5:16757. doi: 10.1038/srep16757 (PMC4645157; doi:10.1038/srep16757)
Supplement: Supplementary Information [file srep16757-s1.pdf]

Supplementary Information

Title: A Deep Look Into Erionite Fibres: an Electron Microscopy Investigation of their Self-Assembly

Authors: Roberto Matassa,[1]* Giuseppe Familiari,[1] Michela Relucenti,[1] Ezio Battaglione,[1] Clive Downing,[2] Alessandro Pacella,[3] Georgia Cametti,[4] and Paolo Ballirano[3,5].

[1] Department of Anatomical, Histological, Forensic and Orthopaedic Sciences, Section of Human Anatomy, Sapienza University of Rome, Via A. Borelli 50, 00161 Rome, Italy.
[2] *Centre for Research on Adaptive Nanostructures* and Nanodevices (CRANN), Trinity College Dublin, Dublin 2, Ireland.
[3] Department of Earth Sciences, Sapienza University of Rome, Piazzale A. Moro, 5, I-00185 Rome, Italy.
[4] Institut für Geologie, Universität Bern, Freiestra[illegible]e 3, CH-3012 Bern, Switzerland.
[5] Laboratorio Rettorale Fibre e Particolato Inorganico, Sapienza University of Rome, Piazzale A. Moro, 5, I-00185 Rome, Italy.

Corresponding Author: *roberto.matassa@uniroma1.it*

Supplementary Table 1S. Morphometric analysis of ED, ER, and ER4G samples.

Sample - Figure	Fiber Width (nm)	Fibril Width (nm)	Shape
ED - 1a	680-26	16.0	Low packing ribbon
ER - 1b	1200	20 (flaky)	High packing rod
ER - 1c	1150-500	300-150	Splitted fibers
ER4G - 1d	1000-500	100	High packing rod
ER4G - 1e	1000	50.0	Splitted fibers

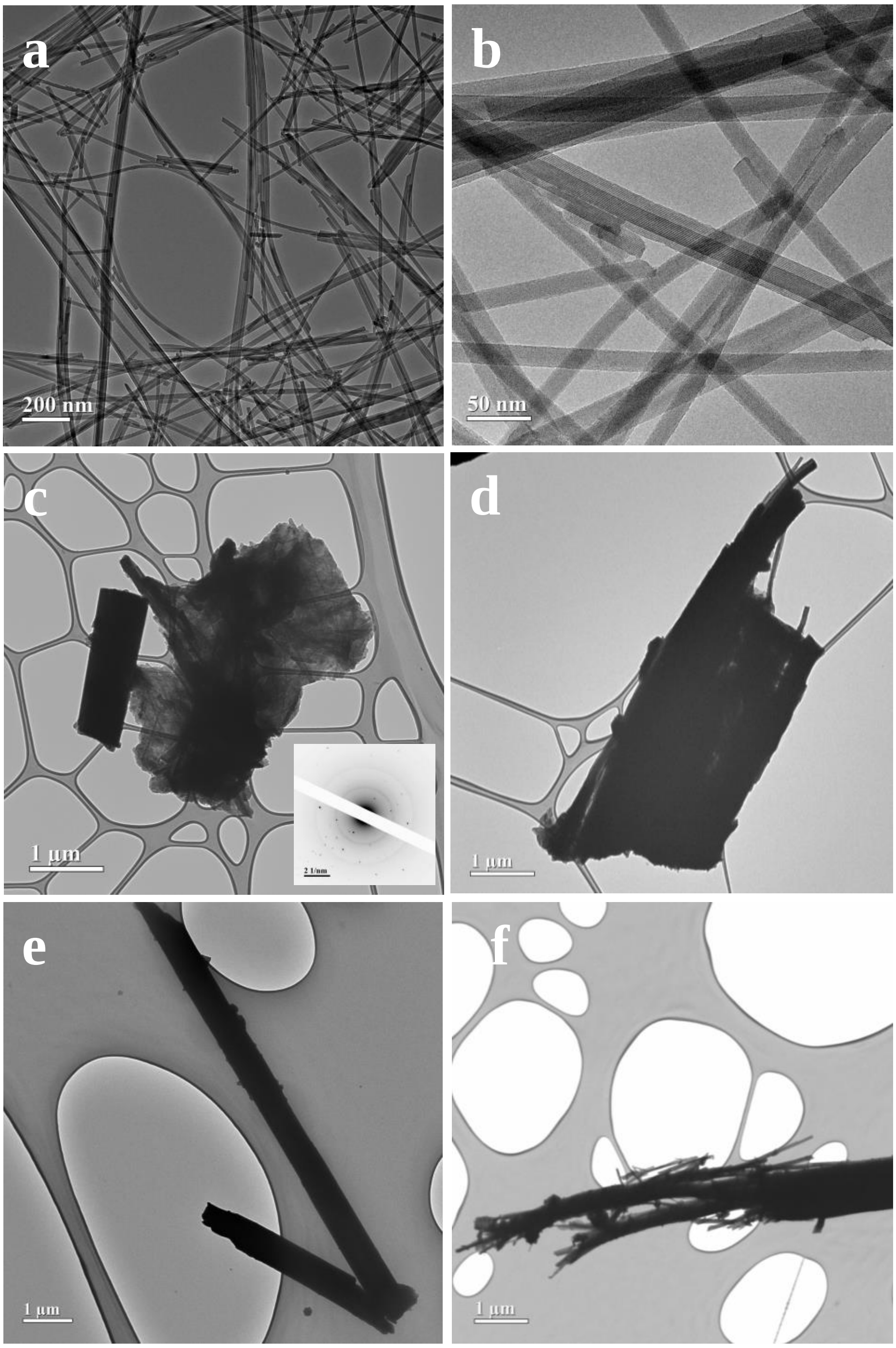

Supplementary Figure S1 Additional representative EM images of ED, ER, and ER4G fibres. (**a**) BF TEM image of woolly aggregates of ED fibres, characterized by different widths, which form small irregular ribbon-like bundles. (**b**) High-magnification BF TEM image exhibiting small overlapped ED fibrils with evident lattice fringes, perpendicular to the long axis of the fibre, corresponding to d_{100} = 1.148 nm. (**c**) BF TEM image of an ER micro-fibre of 0.8 μm in width, with neighbouring nontronite flaky material embedding isolated erionite fibrils with diameters varying from approximately 20 nm to 0.15 μm. Inset: EDP taken from Fig. S1c consisting of the overlapped contribution of a complex array of diffraction spots produced by an erionite fibril and diffraction rings caused by polycrystalline nontronite. (**d**) BF TEM image of ER micro-fibres completely engulfed by a flake of nontronite. Splitted (cleaved) and frayed fibres aggregating into large ones can be noticed around the main micrometric aggregate. (**e**) BF TEM image of ER4G showing two isolated and smooth fibres with 0.60 μm of diameter. (**f**) BF TEM image of ER4G showing splitted micrometric fibres of ca. 1.0 μm in width. The single fibrils belonging to the partially exfoliated bundle have a diameter of ca. 50 nm.

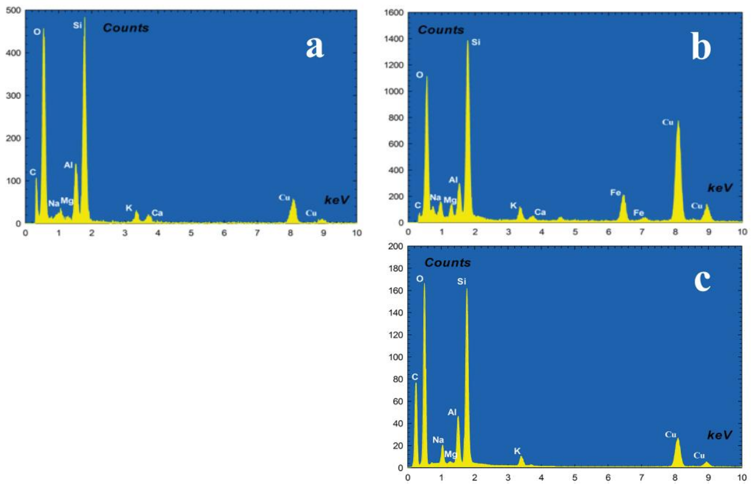

Supplementary Figure S2. EDX analysis of ED and ER. (a) EDX spectral image of ED fibres probed on region II of figure 1a. (b) EDX spectral image of ER fibres probed on figure 1b. (c) EDX spectral image of ER fibres probed on figure 1c.

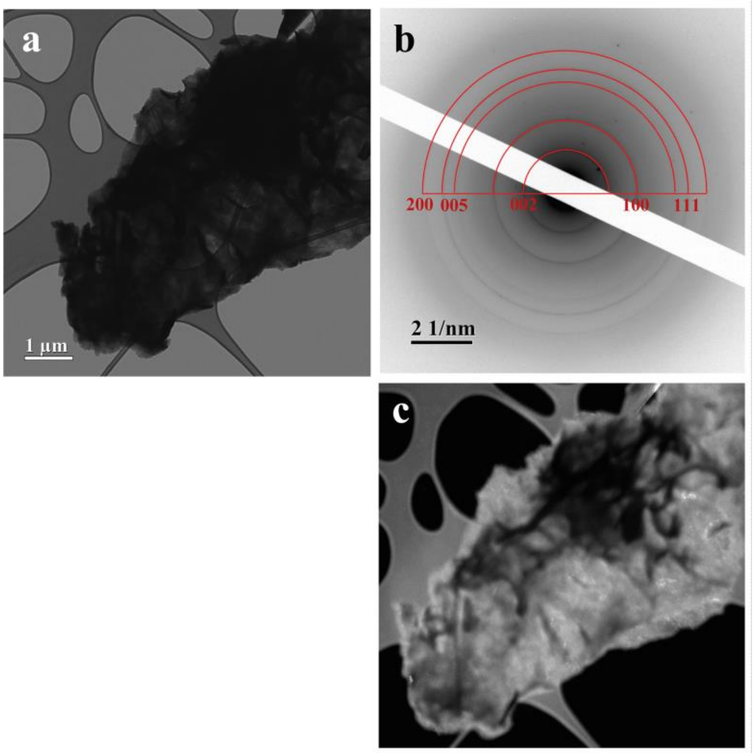

Supplementary Figure S3. Beam damage of ED fibres. (a) BF TEM image of ED fibre aggregates, Inset magnified image of region I. (b) BF TEM image of damaged fibres of region I after a slight increased magnification. Lattice fringes disappear.

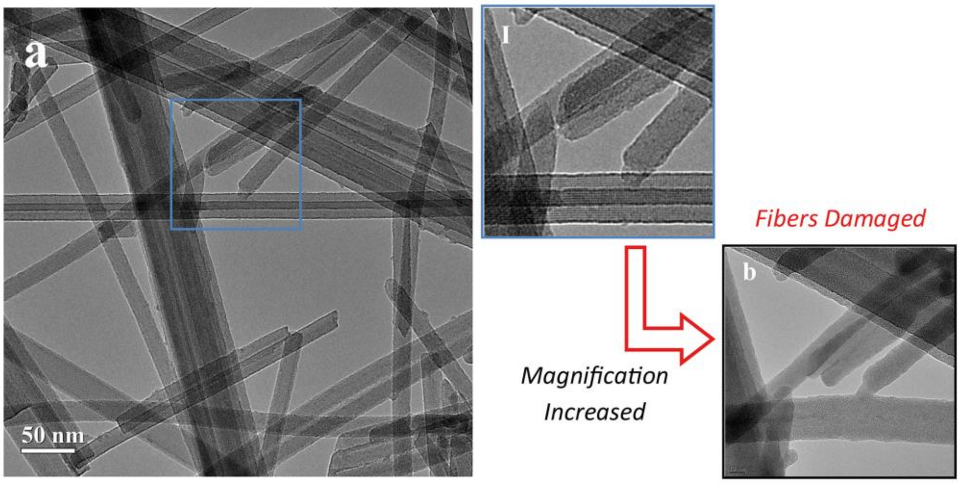

Supplementary Figure S4. Structural studies of nontronite flakes of the ER4G sample. (a) BF TEM image of a nontronite flake. (b) EDP taken from figure S1a showing the Debye rings of nontronite indexed according to PDF 34-0842. (e) DF TEM image of Figure S2a obtained by selecting the micrometric area of the nontronite (002) Debye ring.
